# Mitochondria-DNA copy-number and incident venous thromboembolism among middle-aged women: a population-based cohort study

**DOI:** 10.1007/s11239-021-02446-y

**Published:** 2021-04-15

**Authors:** Peter Nymberg, Ashfaque A. Memon, Jan Sundquist, Kristina Sundquist, Bengt Zöller

**Affiliations:** Center for Primary Health Care Research, Lund University/Region Skåne, Skåne University Hospital Malmö, University Hospital, Jan Waldenströms gata 35, SE-205 02 Malmö, Sweden

## Abstract

Venous thromboembolism (VTE) is the third most common cardiovascular disease. Low amount of mitochondrial DNA copy number (mtDNA-CN) has been associated with arterial cardiovascular disease (CVD) and reflects mitochondrial dysfunctions. However, whether mtDNA-CN is associated with VTE has not been determined. To examine the association between mtDNA-CN and incident VTE among middle-aged women. 6917 women aged 50–64 years, followed for 20 years in the Women’s Health In the Lund Area (WHILA) study. DNA samples for mtDNA quantification were available from 2521 women. Quantification of mtDNA-CN was performed using a well-optimized droplet digital PCR method. After exclusions of women with anticoagulant treatment, women living in nursing homes, and women who were diagnosed with cancer, stroke, VTE, or coronary heart disease at baseline, a cohort of 2117 women remained for analysis. Cox regression was used to analyze the relationship between mtDNA-CN and time to VTE (hazard ratio = HR). In total, 87 women were diagnosed with VTE during follow-up, corresponding to an incidence rate of 2.8 per 1000 person-years. Neither crude nor adjusted HR for mtDNA-CN were significantly associated with incident VTE. A sensitivity analysis with inclusion of excluded women did not change the results. MtDNA-CN was not significantly associated with VTE. The present study suggests that mtDNA-CN, reflecting mitochondrial dysfunction, should not be considered a biomarker that plays a major role for developing VTE. However, due to limited study size we may not exclude minor associations.

**Supplementary Information:**

The online version contains supplementary material available at 10.1007/s11239-021-02446-y.

## Highlights

- This is the first study examining association between mtDNA-CN and incident VTE.
- mtDNA-CN is not associated with incident VTE.
- Low mtDNA-CN is not a major risk factor for VTE among middle-aged women.

## Introduction

There are more than 200,000 new cases of venous thromboembolism (VTE) each year in the United States, with a corresponding mortality rate of about 30% within 30 days [1]. Among VTE survivors, 30% develop recurrent VTE [1] and the increased mortality risk lasts up to 8 years after the first VTE event [1, 2]. Arterial cardiovascular diseases (CVD) share several risk factors with VTE such as obesity, cancer, infections, oestrogens [3], smoking, and obstructive sleep apnea syndrome [4–6]. In addition to the shared risk, CVD and VTE predispose for each other, i.e., prevalent CVD increases the risk of incident VTE [7, 8]. Obesity, with an increased waist circumference, is also a risk factor for metabolic syndrome [9], which increases the risk for varicose veins, VTE, CVD, and diabetes type II [1, 10–14]. According to a systematic review [15] with a meta-analysis of the result from five articles with a total of 8252 cases and 20,904 controls, low amount of mitochondria DNA copy number (mtDNA-CN) is a suggested risk indicator for CVDs. In previous studies, individuals with high BMI and metabolic syndrome have shown a low amount of mtDNA-CN compared with healthy individuals [16, 17]. MtDNA-CN is also suggested to be a biomarker for aging where those with age-related diseases have lower amount of mtDNA-CN [18]. Poor self-rated health has been reported to be associated with lower levels of mtDNA-CN [19]. Poor self-rated health is also a predictor for incident CVD [20]. Lifestyle may also affect the levels of mtDNA-CN; current smoking is associated with lower amount of mtDNA-CN [21] and studies have suggested that high alcohol consumption may affect the levels of mtDNA-CN [22, 23]. In contrast, healthy lifestyle, such as regular physical activity, is associated with higher mtDNA-CN [24, 25]. Physical activity, in accordance with WHO recommendations, lowers risk for both CVD [26] and VTE [27, 28].

Since mtDNA-CN has been associated with CVD, and there are common risk factors that affect the amount of mtDNA-CN shared between both CVD and VTE, we hypothesize that low amount of mtDNA-CN also is associated with an increased VTE risk.

The main aim of this cohort study was to examine the association between baseline mtDNA-CN in middle-aged women (50–64 years) and incident VTE.

## Materials and methods

### Study population

The Women´s Health In the Lund Area study (WHILA) is a prospective cohort study that was conducted in southern Sweden. Sample characteristics, data collection and clinical definitions for WHILA have been described previously [29, 30]. Briefly, the study invited all women (n = 10,766) living in any of the five municipalities around the city of Lund by December 1995, and who were born between December 1935 and December 1945, to a screening procedure that took place from December 1995 until February 2000. A total of 6917 women consented to participate in the study, of which 6916 had complete datasets. Blood samples for DNA analyses were available at baseline only, and were collected midway through the study and therefore were available only for the last 3062 included women. Out of the 3062 blood sampled women, there were 541 samples with poor quality DNA as observed during droplet digital PCR analysis of reference gene. Thus, only 2521 women with sufficient quality of DNA were analyzed. These 541 women was excluded from analysis. Women (n = 13) treated with anticoagulants at baseline were also excluded (ATC code; B01AA03, B01AA01, and B01AB02). Women with prevalent VTE (defined below) or any of the following prevalent diagnoses at baseline were excluded: Stroke [ICD-7; 331, 332, 334.09. ICD-8; 430–434, 436. ICD-9; 430–434, 436. ICD-10; I60-I64 (not I63.6)]; Coronary heart disease (CHD) (ICD-7; 420. ICD-8; 410–414. ICD-9; 410–414. ICD-10; I20-I25.); and Cancer (ICD-7; 140–209). In the cohort, there were 22 women with prevalent stroke, 26 women with prevalent CHD, 232 women with prevalent cancer, and 120 women with VTE before baseline (self-reported and/or by register). Moreover, 14 women who were living in nursing homes were excluded. Some of the women had more than one reason for exclusion, which is why the numbers do not add up. After exclusions, a total of 2117 women remained of the 2521 women with sufficient quality DNA. After exclusions of prevalent stroke, prevalent CHD, prevalent cancer and prevalent VTE, 126 women remained with incident VTE. Of these, 39 women with VTE were censored in the cox regression due to incident CHD, stroke, or cancer before VTE during follow-up. Thus, after censoring for 39 incident cases of stroke, CHD, and cancer, we had 87 incident VTE cases in total.

The cohort was identified via the Swedish population registry that comprised all inhabitants in the Lund area. A health-screening program included laboratory examinations, blood samples and a basic baseline questionnaire that was mailed along with the invitation and collected in conjunction with the first examination. The baseline questionnaire included 104 questions concerning education, household, working status, perimenopausal status, medical history, drug treatment, personal and family history of diabetes or cardiovascular disease (myocardial infarction, stroke, deep venous thrombosis or hypertension in parents or siblings with an event before the age of 60 years). It also contained questions about habits like smoking, alcohol consumption, physical activity, general dietary habits, quality of life, as well as subjective physical and mental symptoms. This questionnaire was a composite of several validated questionnaires.

### Laboratory measurement of mtDNA-CN

The mtDNA-CN was determined by droplet digital PCR (ddPCR) based method for quantification of absolute copy number of mtDNA in whole blood, as described in detail [31]. Compared to real-time PCR, ddPCR has greater precision and improved reproducibility and provides ultrasensitive and absolute nucleic acid quantification [32]. The used ddPCR method for mtDNA determination has been well-optimized with intra- and inter-assay coefficient variances as 3.1% and 4.2% respectively [31].

### Predictor variables

Self-rated health (SRH) was assessed only at baseline from a single question, in which the participants were asked to rate their perceived health in a 7-graded Likert scale from “Very poor” to “Excellent, could not be better” (1 = very poor, 7 = Excellent). In this study, alternative 1–4 was classified as poor SRH and 5–7 as good SRH. Weight and height were rounded off to the nearest 0.1 kg and 0.5 cm. Body mass index (BMI) was calculated as weight in kilograms divided by height in meters squared (kg/m^2^). Varicose veins were defined by ICD-7; 460, ICD-8; 454, ICD-9; 454, and ICD-10; I83. Women were categorized as current smokers (i.e., those who smoked regularly or occasionally), non-smokers and former smokers. Non-alcohol drinkers were defined as not drinking any alcohol (0 g/week), normal consumption more than 0–12 g/week, and high consumption as over 12 g/week. Low physical activity, i.e., less than 30 min vigorous activity 5 days a week [33] was defined by the three lowest answering alternatives (i.e., very low, low and light activity) (Table 1). Overall diet was defined by questions about intake of fat, sugar, fruit, or dietary fiber. If high intake of sugar or fat or low intake of dietary fiber or fruit were reported, the diet was classified as less healthy [29]. If low intake of sugar and fat and high intake of fruit and dietary fiber was reported, the diet was classified as healthy. Education was categorized into three groups: comprehensive school (9 years), upper secondary school (12 years), and university degree.

**Table 1:** Comparison between baseline characteristics between those with incident VTE and those without incident VTE

Baseline measures	Incident VTE	No incident VTE	P
Age^*^			0.34
Mean	57.1	56.9	
Std dev	2.8	2.8	
n	126	1991	
mtDNA^*^			0.49
Mean	116.7	118.4	
Std dev	28.9	27.0	
n	126	1991	
SBP (mmHg)^*^			0.84
Mean	132.3	132.0	
Std dev	14.9	17.2	
n	126	1990	
DBP (mmHg)^*^			0.46
Mean	85.7	85.1	
Std dev	8.5	9.0	
n	126	1991	
Height mean^*^ (cm)	165.6	165.0	0.20
Std dev	5.6	5.4	
n	122	1948	
Weight^*^ (kg)			**0.01**
Mean	70.8	67.9	
Std dev	11.4	10.9	
n	114	1873	
Body mass index			**0.01**
Mean^*^	26.3	25.5	
Std dev	4.0	4.0	
n	126	1991	
Waist (cm)^*^			**0.01**
Mean	84.2	81.5	
Std dev	10.1	10.3	
N	125	1962	
Hip (cm)^*^			**0.02**
Mean	105.8	103.7	
Std dev	7.5	8.0	
N	125	1962	
Waist Hip Ratio			0.11
Mean^*^	0.79	0.78	
Std dev	0.07	0.06	
n	126	1989	
Smoker^**^			0.40
No	73 (59.4%)	1161 (59.5%)	
Current	28 (22.8%)	366 (18.7%)	
Former	22 (17.9%)	426 (21.8%)	
Alcohol^**^			0.54
0 g/w	27 (23.1%)	468 (24.8%)	
> 0–12 g/w	77 (65.8%)	1154 (61.1%)	
> 12 g/w	13 (11.1%)	267 (14.1%)	
Education^**^			0.65
7–9 years	35 (28.0%)	489 (25.0%)	
10–12 years	51 (40.8%)	875 (44.7%)	
> 12 years	39 (31.2%)	595 (30.4%)	
Marital^**^			0.90
Married	99 (79.2%)	1538 (77.6%)	
Unmarried	6 (4.8%)	89 (4.5%)	
Divorced	15 (12.0%)	244 (12.3%)	
Widowed	5 (4.0%)	110 (5.6%)	
Low activity^**^	69 (56.1%)	1093 (55.3%)	0.97
High activity	54 (43.9%)	850 (43.7%)	
Sugar^***^			0.74
Daily	8 (6.5%)	60 (3.1%)	
Sometimes	78 (62.9%)	1326 (67.3%)	
Avoids	38 (30.7%)	584 (29.6%)	
Fat in food^***^			0.25
Much	8 (6.7%)	125 (6.7%)	
Careful with	80 (67.2%)	1154 (61.5%)	
Avoids	31 (26.0%)	597 (31.8%)	
Dietary fiber in food^***^			0.29
Low intake	1 (0.8%)	21 (1.1%)	
Regularly	79 (64.8%)	1164 (59.5%)	
High intake	42 (34.4%)	773 (39.5%)	
Fruit^***^			0.38
Much fruit	83 (66.4%)	1229 (62.2%)	
Eats regularly	39 (31.2%)	708 (35.8%)	
Eats rarely	3 (2.4%)	39 (21.97%)	
Overall diet^***^			0.35
Less healthy	16 (12.8%)	202 (10.2%)	
Healthy	109 (87.2%)	1779 (89.8%)	
Self-rated health^***^			0.49
1. Very poor	1 (0.8%)	8 (0.4%)	
2	0 (0.0%)	30 (1.5%)	
3	5 (4.0%)	101 (5.2%)	
4	19 (15.3%)	271 (13.8%)	
5	37 (28.8%)	496 (25.3%)	
6	38 (30.7%)	5589 (30.1%)	
7. Excellent	24 (19.4%)	463 (23.7%)	
Self-rated health group^***^			0.84
Poor	25 (20.2%)	410 (20.1%)	
Good	99 (79.8%)	1548 (79.1%)	
Amount of food^***^			0.60
Big portions	5 (4.4%)	129 (7.0%)	
Regularly	81 (71.7%)	1186 (64.5%)	
Small portions	27 (23.9%)	525 (28.5%)	
Diabetes before baseline^***^			0.59
Yes	1 (0.8%)	27 (1.4%)	
No	124 (99.2%)	1941 (98.6%)	
Hypertension^**^			0.75
Yes	23 (18.4%)	343 (17.3%)	
No	102 (81.6%)	1639 (82%)	
Varicose veins^***^			0.15
Yes	5 (4.0%)	41 (2.1%)	
No	121 (96.0%)	1950 (98.0%)	
Acetylsalicylic treatment^***^			0.99
Yes	1 (0.8%)	16 (0.8%)	
No	125 (99.2%)	1975 (99.2%)	
Knowledge about family history^**^			0.23
Yes	16 (12.9%)	180 (9.3%)	
No	96 (77.4%)	1622 (83.4%)	
Do not know	12 (9.7%)	144 (7.4%)	

Exclusions: Poor quality mtDNA-CN, prevalent VTE, prevalent CHD, prevalent stroke, prevalent cancer, those medicated with anticoagulant, and those living in nursing homes

P-values were calculated with two-sided Student´s t-test^*^ for continuous variables, with Chi2-test** for normal distributed categorical variables, and with Wilcoxon rank sum test*** for not normally distributed categorical variables between incident VTE and no incident VTE

Bold numbers indicate P values < 0.05

Physical activity was dichotomized into Low activity (Non, Mostly sitting, and Light activity) and High activity (1–2 h/week, regularly and Very high). Self-rated health was dichotomized into the variable Poor self-rated health, with group 1–4 as poor self-rated health and 5–7 as good self-rated health

Information about diseases and medication was obtained from the Swedish Hospital Discharge Register, the Hospital Outpatient Register and the Swedish Cancer Register in addition to self-reported data from WHILA study baseline questionnaires and measurements.

### Outcome variable (VTE)

The definition of VTE includes deep venous thrombosis (DVT), pulmonary embolism (PE), and other VTE types by the following ICD-codes from the Swedish Hospital Discharge Register and the Hospital Outpatient Register:

DVT (deep venous thromboembolism): ICD-7 463, 682. ICD-8 451, 671 (not 671.00). ICD-9 451 (not 451A) 671D 671E. ICD-10 I80 (not I80.0) O22.3, 0871. PE (pulmonary embolism): ICD-7 465, 684. ICD-8 451, 671 (not 671.00). ICD-9 415B, 416 W, 639G, 673C. ICD-10 I26, O88.2, O082. Other VTE types: ICD-7 464, 466, 583.00, 334.40, 334.50. ICD-8 321, 452,453. ICD-9 437G, 451A, 452,453,671C, 671F,6171X. ICD-10 I63.6, I676, I800, I81, I82, O222, O225, O229, O870, O873, O879, O087.

### Statistical analysis

Levels of mtDNA-CN were used as a dichotomized variable according to median, though mtDNA-CN was also used as a continuous variable in the analysis. In the multivariate models, waist circumference was used instead of BMI; this was due to previous studies showing waist circumference as a valid predictor for VTE [34]. A power analysis revealed that a risk ratio of 1.8 could be detected at a significance level of 5% (two-sided) with a power of 82% in the studied population when using mtDNA-CN as a dichotomous predictor variable (https://www.openepi.com/Power/PowerCohort.htm). P-values were calculated with two-sided Student’s t test for continuous variables, with χ^2^-test for dichotomized and normal distributed categorical variables, and Wilcoxon rank sum test for not normally distributed categorical variables. Cox proportional hazards regression was used to analyze the relationship between different variables and time to VTE. Hazard ratios (HR) with 95% confidence intervals (CI) were calculated. Women who were affected by stroke, CHD, or cancer diagnosis before the VTE during the follow-up time were censored. Well-known risk factors for increased VTE-risk were used as confounding variables in the multivariate analysis. At first, in the multivariable model, there was an adjustment for mtDNA-CN and age. In the second model, smoking, waist circumference, physical activity, and varicose veins were added. Among the categorical variables, the same category was used as reference through all analysis. Regarding smoking, “non-smoker” was used as reference, in physical activity the reference was “low physical activity”, in varicose veins it was “no varicose veins” reference, and in mtDNA-CN median, under the median (< 117) was reference. The mtDNA-CN median and mtDNA-CN as a continuous variable were analyzed separately in the different models by Cox regression using the same adjustments. In the main analysis, we excluded all the patients with prevalent VTE, CHD, stroke, cancer, and women treated with anticoagulants at baseline or living in nursing homes. During follow-up, those with incident CHD, stroke, or cancer before VTE were censored. At the next step, a sensitivity analysis was conducted; the only exclusions in the sensitivity analysis were those with poor quality DNA. All other excluded and censored individuals were included in the sensitivity analysis. P-values < 0.05 were considered significant. Analyses were performed in STATA 16.1.

## Result

Measurements at baseline were compared between women, with and without incident VTE, during follow-up (Table 1). Women with incident VTE had a significantly higher weight (p = 0.01) and BMI (p = 0.01) at baseline, and a significantly greater circumference regarding both waist (p = 0.01) and hip (p = 0.02). There was a greater proportion, among those with incident VTE, who had varicose veins.

### Incident VTE and mtDNA-CN

During the follow-up time of 17.9 years, 87 women were diagnosed with VTE. The sum of follow-up time was 31,073.123 years corresponding to a VTE incident rate at 2.8 (95% CI 2.3–3.4) per 1000 person-years. Unadjusted Cox-regression analysis was conducted (Table 2) to determine any associations between possible risk factors and incident VTE. The univariate Cox-regression showed no significant association between increased mtDNA-CN and lower risk for VTE (HR = 0.99, 95% CI 0.98–1.00, p = 0.13). There was a non-significant decreased risk of VTE with mtDNA-CN over the median (HR = 0.76, 95% CI 0.50–1.17, p = 0.21) compared with mtDNA-CN under median. Among potential VTE risk factors, statistically significant associations were observed with weight (HR = 1.02, 95% CI 1.00–1.04 p = 0.01), waist circumference (HR = 1.02, 95% CI 1.00–1.04, p = 0.01), hip circumference (HR = 1.03 95% CI 1.00–1.06 p = 0.02), and varicose veins (HR = 2.89, 95% CI 1.17–7.13, p = 0.02). A significant association with sugar intake was observed. No significant association was shown with incident VTE and current smokers (HR = 1.30, 95% CI 0.78–2.18, p = 0.32) or former smokers (HR = 0.74, 95% CI 0.41–1.33 p = 0.31). Regarding physical activity (HR = 0.87, 95% CI 0.56–1.34 p = 0.52), there were no significant associations with incident VTE. Likewise, there was not shown any significant associations between incident VTE and self-rated health (HR = 1.74, 95% CI 0.24–12.49 p = 0.58) (Table 2).

**Table 2:** Univariate Cox regression with variables associated with VTE

	Hazard ratio	95% CI	p	n	Failures
mtDNA-CN	0.99	0.99–1.00	**0.013**	2117	87
mtDNA-CN median^*^	0.76	0.50–1.17	0.21	2117	87
Age	1.05	0.97–1.13	0.23	2117	87
Height	1.02	0.99–1.06	0.20	2070	85
Weight	1.02	1.00–1.04	**0.01**	1987	79
BMI	1.18	0.93–1.49	0.18	2117	87
Waist circumference	1.02	1.00–1.04	0.**01**	2087	86
Hip circumference	1.03	1.00–1.05	**0.02**	2087	86
WHR	1.29	0.51–3.15	0.59	2115	87
Diastolic blood pressure	1.01	0.99–1.04	0.30	2117	87
Systolic blood pressure	1.00	0.99–1.02	0.62	2116	87
Education				2084	87
7–9 years	1.39	0.84–2.28	0.20		
10–12 years	Ref				
> 12 years	1.00	0.60–1.67	1.00		
Marital				2106	87
Married	Ref				
Unmarried	1.32	0.53–3.29	0.54		
Divorced	1.07	0.57–2.03	0.83		
Widowed	0.89	0.32–2.44	0.82		
High activity /low activity	0.87	0.56–1.34	0.52	2066	85
Smoking				2076	85
Non smoker	Ref				
Current smoker	1.30	0.78–2.18	0.32		
Former smoker	0.74	0.41–1.33	0.31		
Alcohol				2006	81
0 g/w	0.98	0.59–1.64	0.94		
0–12 g/w	Ref				
> 12 g/w < 130 g/w	0.67	0.32–1.40	0.28		
Sugar				2094	87
Daily	2.87	1.30–6.33	** < 0.01**		
Sometimes	Ref				
Avoids	1.32	0.84–2.08	0.23		
Fat in food				1995	81
Much	1.08	0.47–2.52	0.85		
Careful with	Ref				
Avoids	0.84	0.51–1.38	0.49		
Fiber				2080	84
Low intake	1.23	0.17–8.88	0.84		
Regularly	Ref				
Much	0.96	0.62–1.49	0.85		
Fruit				2101	87
Much fruit	0.96	0.61–1.48	0.84		
Eats regularly	Ref				
Eats rarely	1.16	0.28–4.84	0.84		
Overall diet Less healthy/healthy	1.56	861–2.98	0.14	2106	87
Amount of food				1953	77
Big portions	0.64	0.23–1.77	0.39		
Regularly	Ref				
Small portions	0.64	0.37–1.12	0.12		
Acetylsalicylic	1.83	0.25–13.13	0.55	2117	87
Knowledge about FH^**^ VTE				2070	86
Yes	1.58	0.86–2.92	0.14		
No	Ref				
Do not know	1.17	0.54–2.55	0.69		
Diabetes	0.89	0.12–6.39	0.91	2093	87
Hypertension	1.30	0.78–2.19	0.32	2107	87
Prevalent varicose veins	2.89	1.17–7.13	**0.02**	2117	87
Self-rated health Poor/good	1.74	0.24–12.49	0.58	2117	87

Exclusions: Poor quality mtDNA-CN, prevalent VTE, prevalent CHD, prevalent stroke, prevalent cancer, those medicated with anticoagulant, and those living in nursing homes. Censored for CVD stroke and cancer before VTE diagnosis during follow-up. Self-rated health was dichotomized into the variable Poor self-rated health, with group 1–4 as poor self-rated health and 5–7 as good self-rated health. Bold numbers indicate statistically significant differences

^*^Reference under median

^**^ = family history

In the second step, a multivariable Cox regression analysis was performed, at first when adjusted for age (Table 3) dichotomized mtDNA-CN over median showed a non-significant decreased risk for VTE (HR = 0.76, 95% CI 0.50–1.77 p = 0.22) compared to mtDNA-CN under the median (Table 3). With age adjusted Cox regression and mtDNA-CN as a continuous variable, a non-significant decreased risk was shown with increasing mtDNA-CN (HR = 0.99, 95% CI 0.98–1.00 p = 0.14). The last model 3, adjusted for age, smoking, waist circumference, physical activity, and varicose veins showed mtDNA-CN over the median a non-significant decreased risk of incident VTE (HR = 0.89, 95% CI 0.57–1.38 p = 0.60) using mtDNA-CN under the median as reference. Using the same adjustments but with mtDNA-CN as a continuous variable showed no effect of neither decreased nor increased risk for incident VTE (HR = 1.00, 95% CI 0.99–1.00 p = 0.34) (Table 3).

**Table 3:** Cox regression with mtDNA-CN, analyses with median and continuous were done in separate models

Model	1	2	3
Total n	2117	2117	2003
Failures n	87	87	82
mtDNA median^*^			
HR	0.76	0.76	0.89
CI	0.49–1.17	0.50–1.17	0.57–1.38
P	0.21	0.22	0.60
mtDNA (continuous)			
HR	0.99	0.99	1.00
CI	0.98–1.00	0.98–1.00	0.99–1.00
p	0.13	0.14	0.34
	Model 1 mtDNA-CN	Model 2 mtDNA-CN and age	Model 3 mtDNA-CN, age, smoking, waist circumference physical activity and prevalent varicose veins

Exclusions: Poor quality mtDNA-CN, prevalent VTE, prevalent CHD, prevalent stroke, prevalent cancer, those medicated with anticoagulant, and those living in nursing homes. Censored for CVD stroke and cancer before VTE diagnosis during follow-up

^*^Reference is mtDNA-CN under the median

### Sensitivity analysis

The only exclusions done in the sensitivity analysis were those with poor quality DNA (541 women). In the sensitivity analyses, a total of 2521 women were remaining and out of these, 142 women were affected with incident VTE during follow-up. Moreover, in the sensitivity analyses, no censoring was performed for women with incident stroke, CHD, or cancer. The sum of follow-up time in the sensitivity analysis was 38,805.318 years corresponding to a VTE incident rate at 3.7 (95% CI 3.1–4.3) per 1000 person-years total analysis time at risk under observation. The result of the univariate sensitivity analysis (Supplementary Table 1) is approximately the same as the univariate main analysis regarding hazard ratios and confidence intervals. The same variables showing significant association in main analysis were significant in the sensitivity analysis, except for knowledge of family history of VTE, which showed an association with incident VTE in the sensitivity analysis (HR = 1.67, 95% CI 1.05–2.68, p = 0.03) (Supplementary Table 1). Regarding varicose veins, it was the opposite with no significant associations in the sensitivity analysis (Supplementary Table 1). The multivariable sensitive analysis (Table 4) showed the same pattern as the main multivariable analysis. There were minor differences in hazard ratios and level of significance between the main analysis and the sensitivity analysis.

**Table 4:** Sensitivity analysis cox regression with mtDNA-CN, analyses with median and continuous were done in separate models

Model	1	2	3
Total n	2401	2401	2277
Failures n	142	142	136
mtDNA median^*^			
HR	0.87	0.87	0.94
CI	0.63–1.21	0.63–1.22	0.67–1.31
p	0.41	0.42	0.74
mtDNA (continuous)			
HR	1.00	1.00	1.00
CI	0.99–1.00	0.99–1.00	0.99–1.01
p	0.40	0.41	0.66
	Model 1 mtDNA-CN	Model 2 mtDNA-CN and age	Model 3 mtDNA-CN, age, smoking, waist circumference physical activity and prevalent varicose veins

The only exclusions were those with poor quality mtDNA-CN

^*^Reference is mtDNA-CN under the median

## Discussion

The present first study on mtDNA-CN and future VTE risk found no significant association between mtDNA-CN and incident VTE. Based on our results, we suggest that mtDNA-CN should not be considered a biomarker that plays a major role for developing VTE. VTE and arterial CVD share many risk factors, but some are unique to VTE or CVD [3]. The mtDNA-CN appears to be a factor that is only associated with CVD [15] and not VTE. Thus, mitochondrial dysfunction reflected by low copy number of mtDNA appears not to be of similar importance in VTE as in arterial CVD [15]. Due to limited study size, we may not exclude minor associations. The level of mtDNA-CN is determined by heredity, in addition to the influence on one's lifestyle habits such as physical activity, smoking, and alcohol consumption [21–25, 35]. Poor self-rated health is also associated with low levels of mtDNA-CN [19]. Factors like these, for instance smoking and physical activity, are important for the development of arterial CVD [26]. Nevertheless, we found no significant association between VTE and mtDNA-CN.

An association between incident VTE and dietary habits has previously been reported [36] where a diet with low amount of red meat and high amount of grains and vegetables lowers the risk of incident VTE. Although, in our study, only self-reported daily consumption of sugar showed a significantly increased risk of VTE in this cohort. High sugar intake is a well-known dietary habit that increases the risk of arterial CVD and might also affect VTE risk [37, 38]. However, we think the association with sugar consumption and VTE in the present study is a chance finding. A previous study of the same cohort containing all 6917 women did not show any significant associations between daily sugar consumption, other dietary habits or any significant association between physical activity and incident VTE [30]. In fact, diet remains to be a proven risk factor for VTE [39].

A limitation of the study is the study size. However, well-established risk factors such as anthropometric measures for overweight (weight, BMI, waist circumference, and waist circumference) and varicose veins were all associated with future VTE risk. Even if increasing age is a well-known risk factor for VTE [40, 41], no association between increasing age and incident VTE was found, most likely due to the narrow age span between 50 and 60 years of age at baseline. The method used for measuring physical activity in this study can affect the results, related to the known risk of overestimating the time in physical activity when self-reported [42, 43], which is another limitation of the study regarding physical activity. Another limitation is the inclusion of women only. However, there is no hypothesis suggesting that the importance of mtDNA-CN should be sex dependent. Still, the copy number of mitochondrial DNA has been reported to be lower in males compared with females both in humans [44, 45] and in Drosophila melanogaster [45, 46]. It will therefore be of interest to investigate whether mtDNA-CN is associated with VTE among males.

A strength of the study is the population-based cohort design with long follow-up time. Using a hospital-based diagnosis of VTE is another strength. The Swedish Hospital register has a general validity between 85 and 95% [46]. VTE has been validated with a 95% positive predictive value [47]. In Sweden, VTE is usually confirmed with objective methods [48–50].

Several common risk factors [3–5] for both VTE and CVDs have been described, and mtDNA-CN has been shown to be a risk indicator of CVDs [15]. Although, mtDNA-CN does not to appear to be a biomarker that is associated with VTE.

## Conclusion

MtDNA-CN was not associated with incident VTE among middle-aged women. MtDNA-CN is suggested not to be of major importance for the development of VTE.

### Strengths and limitations

This is the first study aiming to investigate the association between mtDNA-CN and incident VTE, which is a strength of this study. Performing the study in a small cohort containing only middle-aged women with a narrow age span is a limitation.

## Supplementary Information

Below is the link to the electronic supplementary material.

Supplementary file1 (DOCX 21 KB)

## Abbreviations

VTE: Venous thromboembolism
CVD: Arterial cardiovascular disease
mtDNA-CN: Mitochondrial DNA
PE: Pulmonary embolism
DVT: Deep vein thrombosis
SBP: Systolic blood pressure
DBP: Diastolic blood pressure
WHILA: Women´s Health In the Lund Area study
ddPCR: Optimized droplet digital PCR
SRH: Self-rated health
BMI: Body mass index
HR: Hazards ratio
FH: Family history
