# Supplementary material for: Mitochondria-DNA copy-number and incident venous thromboembolism among middle-aged women: a population-based cohort study
Source: J Thromb Thrombolysis. 2021 Apr 15;52(1):148–57. doi: 10.1007/s11239-021-02446-y (PMC8282550; doi:10.1007/s11239-021-02446-y)
Supplement: Supplementary file 1 — Supplementary file1 (DOCX 21 KB) [file 11239_2021_2446_MOESM1_ESM.docx]

| Supplementary Table 1. Sensitivity analysis using univariate Cox regression with variables associated with VTE. Only exclusions were those with poor mtDNA-CN | | | | | |
| --- | --- | --- | --- | --- | --- |
| **Baseline variables** | **Hazard ratio** | **95% CI** | **p** | **n** | **Failures** |
| mtDNA | 1.00 | .99-1.00 | .40 | 2401 | 142 |
| mtDNA under/over the median | 0.87 | .63-1.24 | .41 | 2401 | 142 |
| Height | 1.02 | .99-1.05 | .23 | 2346 | 138 |
| Weight | 1.02 | 1.00-1.03 | **.02** | 2261 | 130 |
| BMI | 1.18 | .98-1.42 | .08 | 2401 | 142 |
| Waist circumference | 1.02 | 1.00-1.03 | **<.01** | 2368 | 141 |
| Hip circumference | 1.02 | 1.00-1.04 | **.03** | 2368 | 141 |
| Baseline WHR | 1.48 | .69-3.17 | .31 | 2399 | 142 |
| Diastolic blood pressure | 1.00 | .98-1.02 | .82 | 2401 | 142 |
| Systolic blood pressure | 1.00 | .99-1.01 | .75 | 2400 | 142 |
| Education  7-9 years 10-12 years > 12 years | 1.27  Ref  1.13 | .85-1.90  .76-1.67 | .24  .56 | 2365 | 141 |
| Marital Married Unmarried Divorced Widowed | Ref  1.00  1.07  .78 | .44-2.28  .66-1.74  .34-1.77 | 1.00  .79  .55 | 2390 | 141 |
| High activity /Low activity | .95 | .68-1.33 | .77 | 2344 | 139 |
| Smoking  Non smoking  Former  Current | Ref  .79  1.17 | .51-1.24 .78-1.79 | .30 .44 | 2358 | 139 |
| Alcohol  0g/w  0-12g/w >12g/w<130g/w | .92  Ref  .78 | .61-1.38  .45-1.34 | .68  .36 | 2273 | 131 |
| Sugar daily  sometimes  avoids | 2.00  Ref  1.17 | .97-4.12  .81-1.67 | .06  .40 | 2371 | 140 |
| Fat in food Much  careful with Avoids | 1.13  Ref  .82 | .59-2.17  .56-1.21 | .72  .32 | 2251 | 134 |
| Fiber  Low intake  regularly  Much | 1.28  Ref  .73 | .32-5.20  .51-1.05 | .73  .08 | 2359 | 138 |
| Fruit  Much fruit  Eats regularly  Eats rarely | 1.12  Ref  1.25 | .79-1.60  .39-4.00 | .53  .71 | 2381 | 141 |
| Overall diet Less healthy vs  Healthy | 1.43 | .88-2.31 | .15 | 2390 | 141 |
| Amount of food Big portions Regularly Small portions | 0.78  Ref  .84 | .36-1.68  .56-1.27 | .52  .41 | 2210 | 127 |
| Acetylsalicylic | .48 | .07-3.42 | .46 | 2401 | 142 |
| Knowledge about FH* VTE  Yes  No  Do not know | 1.68  Ref  1.26 | 1.05-2.69  .69-2.29 | **.03**  .45 | 2348 | 140 |
| Diabetes | 2.26 | .32-16.16 | .42 | 2342 | 140 |
| Hypertension | 0.97 | .63-1.49 | .88 | 2390 | 141 |
| Prevalent varicose veins | 1.86 | .82-4.22 | 0.14 | 2401 | 142 |
| Self-rated health | 1.01 | .32-3.18 | .98 | 2401 | 142 |
| Self-rated health was dichotomized into the variable Poor self-rated health, with group 1-4 as poor self-rated health and 5-7 as good self-rated health, *= family history | | | | | |
